# Protein subcellular localization prediction tools^☆^

**DOI:** 10.1016/j.csbj.2024.04.032

**Published:** 2024-04-15

**Authors:** Maryam Gillani, Gianluca Pollastri

**Affiliations:** School of Computer Science, University College Dublin (UCD), Dublin, D04 V1W8, Ireland

**Keywords:** Subcellular localization predictions, Machine learning/deep learning, Protein predictions, Bioinformatics

## Abstract

Protein subcellular localization prediction is of great significance in bioinformatics and biological research. Most of the proteins do not have experimentally determined localization information, computational prediction methods and tools have been acting as an active research area for more than two decades now. Knowledge of the subcellular location of a protein provides valuable information about its functionalities, the functioning of the cell, and other possible interactions with proteins. Fast, reliable, and accurate predictors provides platforms to harness the abundance of sequence data to predict subcellular locations accordingly. During the last decade, there has been a considerable amount of research effort aimed at developing subcellular localization predictors. This paper reviews recent subcellular localization prediction tools in the Eukaryotic, Prokaryotic, and Virus-based categories followed by a detailed analysis. Each predictor is discussed based on its main features, strengths, weaknesses, algorithms used, prediction techniques, and analysis. This review is supported by prediction tools taxonomies that highlight their rele- vant area and examples for uncomplicated categorization and ease of understandability. These taxonomies help users find suitable tools according to their needs. Furthermore, recent research gaps and challenges are discussed to cover areas that need the utmost attention. This survey provides an in-depth analysis of the most recent prediction tools to facilitate readers and can be considered a quick guide for researchers to identify and explore the recent literature advancements.

## Graphical Abstract

**Figure:**
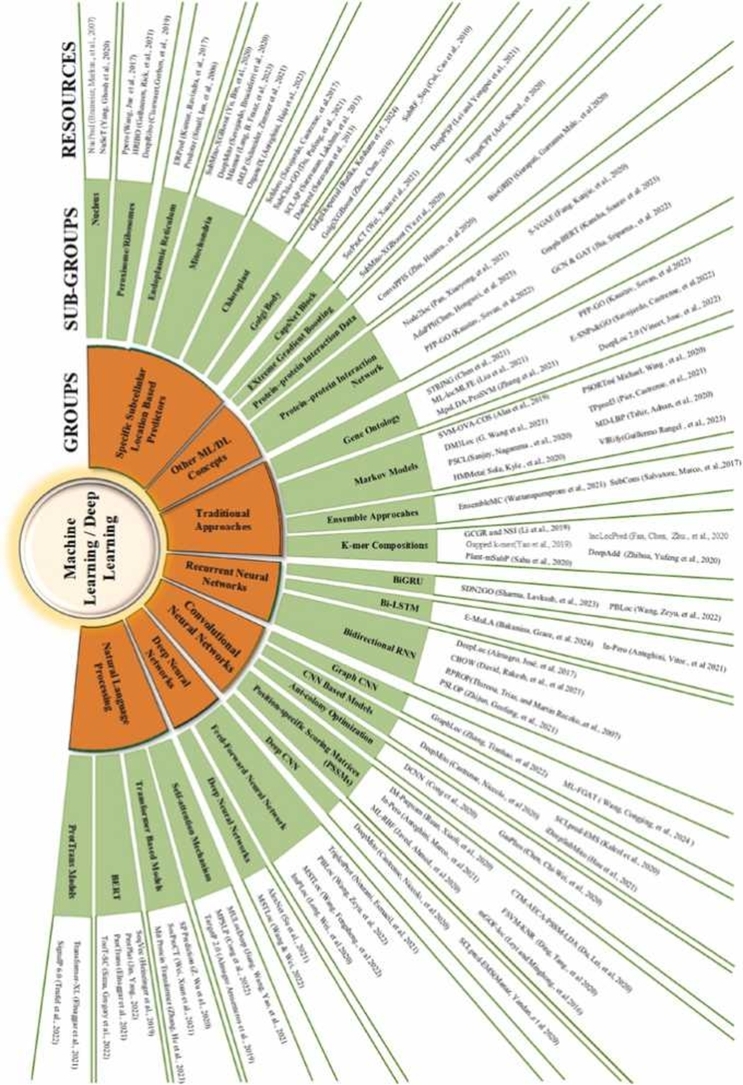

## 1 Introduction

Proteins are complex molecules. They are comprised of long chains of amino acids that perform a variety of functions in different organisms. The function of a protein depends on the compartment or organelle where it is located. Each subcellular compartment has a well-defined function within a cell that has a distinct physicochemical environment. Physic- ochemical properties drives the proper functioning of the proteins [1]. Also, it is essential for proteins to be destined for their specific locations or compartments to perform their functions [2]. Knowledge of proteins in different organelles and their subcellular locations is essential to gain insight into how the cell functions as the primary unit of life [3].

Experimental methods to explore the knowledge of pro- teins in different organelles and their subcellular locations are relatively expensive, labour-intensive and time-consuming process. Due to this, a large informational gap exists be- tween known proteins and their location information [4]. Alternately, computational subcellular location and predic- tion tools have the advantage of being cost-effective, time- efficient, and capable of complementing resource-consuming experimental techniques adequately [5]. During the past decade, researchers have been working considerably to de- sign computational tools and techniques to explore various aspects of location information and subcellular localization predictions.

Computational methods that are widely used in subcel- lular localization prediction tools are roughly divided into; Sequence-based methods, Annotation/ Information/ Knowl- edge/ Homology-based methods, and Structure-based meth- ods [6]. Sequence-based predictors make use of known sort- ing signals, amino acid composition information, or often- times both [7]. Whereas, annotation-based predictors make use of information about functional domains and motifs, protein-protein interactions, homologous proteins, annotated Gene Ontology (GO) terms, and textual information pri- marily from SwissProt keywords or PubMed abstracts [8]. These methods are favourable for predicting the functions of interacting proteins and proteins from coexpressed genes but are seriously restricted by the potentially large noise in protein–protein interaction data and the insufficient number of annotated proteins [9]. However, sequence-based methods are widely adopted in protein function prediction due to the relatively easy access of abundant high-quality sequence data in public databases and its significant ability to predict the function of remotely relevant proteins and the homologous proteins of distinct functions [10].

Predicting tools that use experimentally verified annotated protein sequences to predict subcellular locations are known as Homology-based predicting tools or Template-based Pre- dictors [11]. On the other hand, sequence-based methods tend to predict locations based on protein sequences only i.e. Template-free predictions. The disadvantage of homology- based methods is the limited availability of templates of a large number of proteins [12]. Un-annotated proteins pose significant challenges to homology-based methods as they do not have a relevant template to rely on. In such challenging situations, Ab-initio predicting tools provide solutions for subcellular localization predictions. [13].

Sequence-based methods are further categorized into similarity-based methods. Similarity-based methods e.g. re- lying on BLAST [14] and HMMER, typically assign an unannotated protein to the function of another protein similar in sequence to that protein [15]. The main drawback is these tools are heavily dependent on sequence homology and their performance degrades or breaks down completely when only remote or no convincing homology is available. Methods like Support Vector Machines (SVM) [16], [17], and deep neural networks such as Convolutional Neural Networks (CNN) [18], [19], Long Short Term Memory Networks (LSTMs) [20], [21], Recurrent Neural Networks (RNNs) PSLOP [22] are considerably advancing to propose handy solution for non-homologous or template free predictions.

**Fig. 1:**
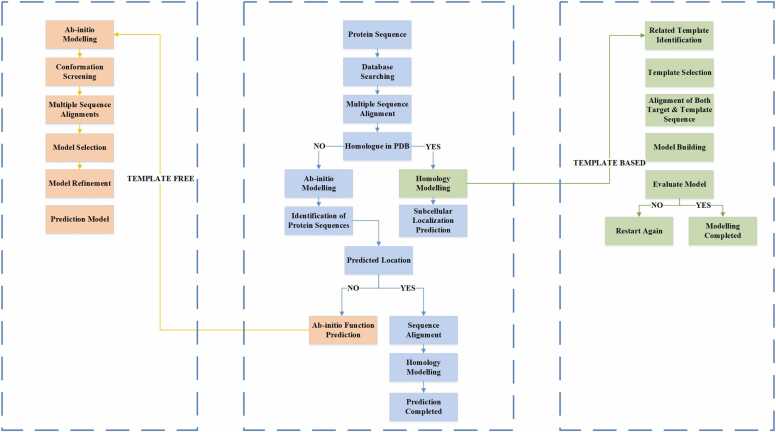
Abstract level step-by-step flowchart for subcellular localization predictions.

A further category is meta-predictors which integrate the prediction results of multiple tools [23], [24]. There are also hybrid methods that combine sequence-based and annotation-based information to harness the advantages of both approaches [25], [26], [27]. Fig. 1 illustrates the flowchart and a step-by-step procedural guide for a machine learning approach for sub-cellular localization prediction along with Template-based as well as Template-free method- ologies.

**Fig. 2:**
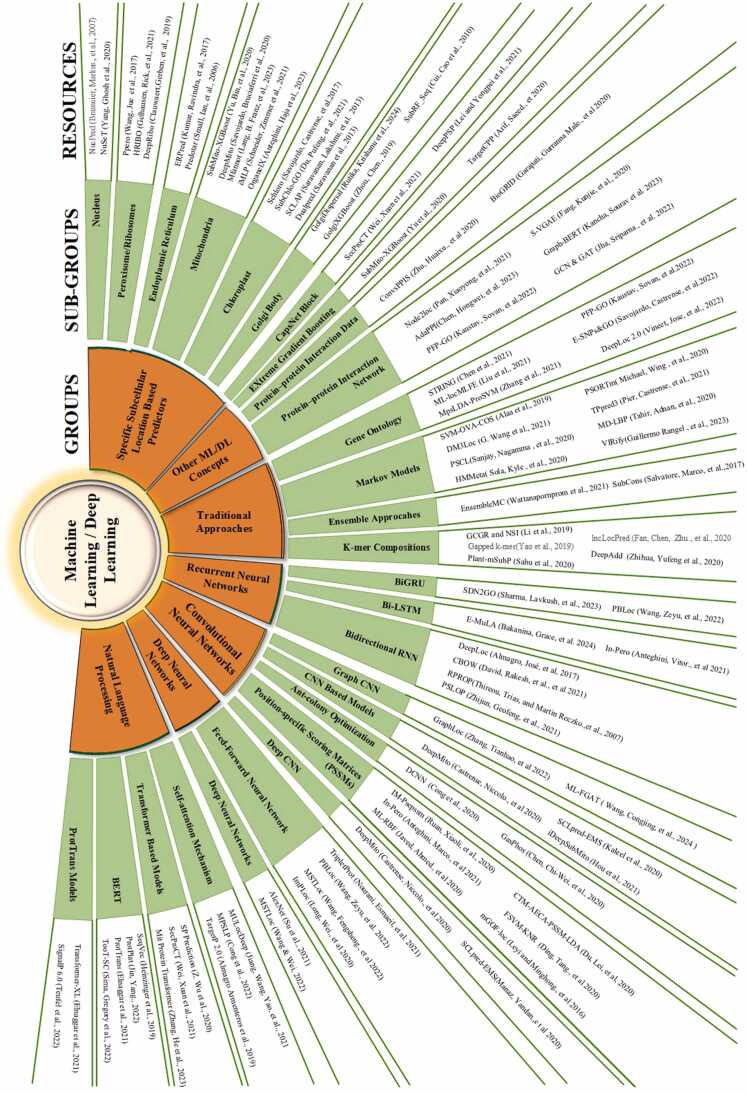
Categories and sub-categories of machine learning and deep learning-based subcellular localization prediction tools.
2) This survey provides two taxonomies for single- category supported predicting tools and multi-category based predicting tools to give a very clear and con- cise illustration that is further distinguished based on colours assigned to the method involved. These tax- onomies are quick guides for users to look for method- ology adapted, predictor class, and relevant tools.
3) As this survey covers broader sets of recent prediction tool categories, it facilitates readers in better under- standing their relationships, features, algorithms, and class of tools in greater detail.
4) Users can select the tools they need based on the infor- mation summarized and can access them through the detailed Table provided. Apart from the applicability of the tools, only actively maintained tools are listed.
5) Another highlighted contribution is to provide a diverse set of research gaps and current challenges that are encountered during the prediction phases in the form of a dendrogram. This dendrogram serves as a reference to many recent research problems and loopholes for users. It provides an excellent kickstart to work on potential solutions.
6) A tool-based information summary table is given that covers several locations and features predicted by a tool followed by methods and models adapted. It is sup- ported by extensive evaluations of subcellular localiza- tion prediction tools while providing detailed insights on why some tools have better prediction accuracy than others.

There are few recent surveys that cover protein subcellular localization predictions tools and techniques [28], [29], [30],

[31], [32], [33], [34]. However, these surveys are either only descriptive or carry a limited domain spectrum with small sets of tools. Also, greater help is taken from textual analysis only which requires readers to go through heavy written content to explore recent predictors. Therefore, this article is established based on three diverse taxonomies and other diagrammatic illustrations to facilitate readers better. In the light of already published literature:

The highlighted contributions of the paper are enumerated as follows:

1) A comprehensive, precise and well-directed machine learning and deep learning-based classification dia- gram is designed that covers 7 primary areas of ML/DL methods followed by 28 sub-classifications. All these classifications are supported by more than 100 recent and dynamic subcellular localization prediction tools. This diagram (Fig. 2) mostly covers 5 to 7 years old diverse tools and techniques of protein subcellular localization.

The rest of the paper is organized as follows: Section II covers categories of subcellular localization prediction tools for both single and multi categories further supported by taxonomic illustrations, Section III covers challenges and re- search constraints, Section IV concludes the paper followed by references used.

## 2 Categories of subcellular localization prediction tools

Protein subcellular localization prediction is essential for revealing biological information and the functioning of the cells [35]. Cells are the basic unit of life and contain many protein molecules located in and operating within different organelles. Proteins in various organelles or subcellular lo- cations have different functionalities [36], and co-location within the same organelle is generally a requirement for cooperation between proteins. Therefore, knowledge of sub- cellular localization plays a significant role in understanding specific functionalities and biological processes [37]. A con- siderable amount of work is ongoing in the bioinformatics research community to expand our knowledge of the subcel- lular localization of proteins. However, complete knowledge is still elusive due to the highly complex nature of proteins and the processes and signals that direct them to different subcellular compartments [4].

Subcellular localization prediction tools are platforms for knowledge discovery via machine learning [38]. Massive amounts of protein data were once considered a complex scenario for subcellular location prediction [39]. Now, ma- chine learning has shifted the paradigm, and access to more data is considered vital for better prediction results. Knowl- edge of proteins in different organelles and their subcellular locations is essential to gain insight into how the cell func- tions as a primary unit of life [40]. During the past decade, researchers have been working rigorously to improve the accuracy of subcellular localization prediction tools. Com- putational prediction tools have the advantage of being cheap and extremely fast to run compared to complex and resource- consuming experimental techniques. Still, the accuracy of their predictions is often an issue [41]. Computational meth- ods that are widely used in subcellular localization predictors may be divided into four main categories; 1) Sequence- based predictors, 2) Annotation-based predictors, 3) Hybrid predictors, 4) Meta-predictors [42], [32].

Sequence-based methods utilize only amino acid se- quences from the query protein as input [43]. They rely on detecting sequence-coded signals such as N-terminal Target- ing Peptides (NTP) [44], [45] or Nuclear Localization Signals (NLS) [46]. Sequence-based predictors consider that the amino acid composition of proteins is correlated with their localization. They are subdivided into two categories, i.e. homology-based, and ab initio approaches [47]. Homology- based methods rely on homologous proteins that are anno- tated for subcellular localization, whereas ab initio methods generally adopt statistical approaches relying on the primary sequence of the query only.

Homology-based methods are also known as annotation- based methods or knowledge-based methods [48]. They are widely based on Gene Ontology (or PubMed abstracts) and Swiss-Prot keywords. Most of them are capable of search- ing/transferring the sequence (if the information is not avail- able for the query protein) from the annotation of close ho- mologs [49]. Because of this, they are generally more accu- rate in their predictions than other known methods. However, the absence of close homologous proteins often degrades the accuracy of their predictions when only remote homologs are available (also known as the twilight zone phenomenon), and prevents them from producing any prediction at all in those cases where no homologs of known location can be found [48].

Hybrid predictors are based on both sequence and homology-based methods [50], [51]. They adapt desired fea- tures from both types of methods for better prediction and results. In other words, hybrid predictors use the detection of sorting signals along with composition information, ho- mology transfer, and whatever additional information may be available [52]. Meta-predictors use other predictors’ results and combine them to deduce suitable predictions. Another aspect differentiating predictors is the number of localization classes they adopt as their targets. Unique subcellular loca- tions may be hundreds, but for many of them, only handfuls of annotated examples are available. Because of this, most systems restrict their predictions to a limited number of well- represented localizations, typically between 2 and a dozen, with some systems only focusing on one task (e.g. secreted from the cell vs. non-secreted) and others adopting multiple categories at the same time [53].

**Fig. 3:**
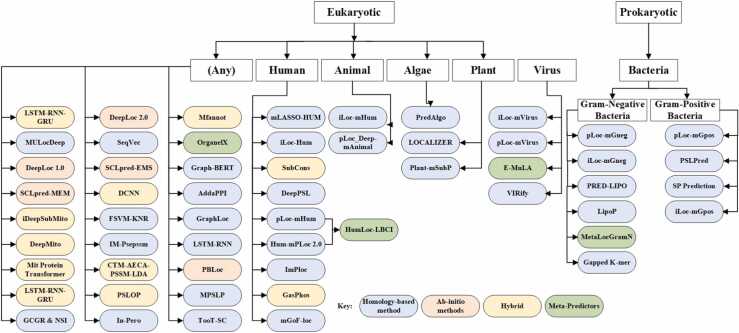
Tools for subcellular localization predictions for single category.

A detailed Taxonomy of subcellular localization predictors based on the four aforementioned criteria is given in Fig. 3 that illustrates tools for single-category predictors only. It in- dicates Hybrid methods in yellow, Meta-Predictors in green, Sequence-based methods in orange, and Homology-based methods in blue. Predicting tools are discussed based on three categories i.e. (Eukaryotes, Prokaryotes, and Viruses). Recent examples of tools are given for each category and their methods are discussed in subsequent sections to help researchers with better predictor choices for protein subcel- lular localization.

### 2.1 Subcellular localization prediction tools for eukaryotes

Eukaryotic Cells are distinguished based on the presence of a membrane-bounded nucleus, organelles, and numerous internal structures such as the Endoplasmic Reticulum, Golgi apparatus, secretory vesicles, etc. Eukaryotes evolved from prokaryotes. They contain a vast and diverse amount of or- ganisms, including Humans, Plants, Animals, Fungi, various kinds of algae, etc. Several subcellular localization predictors for the eukaryotic family have been developed to date. One such example is the Multi-label Least Absolute Shrinkage and Selection Operator (LASSO) for Humans. mLASSO- Hum [54] is a multi-label predictor that provides an inter- pretable solution for large-scale single and multi-label human proteins with the additional feature of providing biological interpretability for the prediction of protein existence at a particular location. mLASSO-Hum also avoids overfitting of the model, unlike existing homology-based predictors that often lack interpretability and suffer from overfitting due to the high dimensionality of feature vectors.

Unlike mLASSO-Hum, various other multi-label predic- tors, e.g. Hum-mPLoc 2.0 [55], mGOASVM [56] HybridGO-Loc [57] R3P-Loc [58] mPLR-Loc [59] Multi-Model Multi- Label Learning [60] Dimensionality Reduction Random Pro- jection [61] KNN-SVM ensemble classifier [62] use Gene Ontology (GO) information to train various categories of statistical algorithms. This may lead to good predicting per- formances, with the drawback of lacking interpretability, i.e. not providing a rationale behind the prediction of a protein residing at a particular location. Depending on the precise type of algorithm and training data adopted, overfitting of the training set may also be an issue. HumLoc-LBCI [41] is quite similar to mLASSO-Hum, except it uses a novel V-dimensional feature vector rather than an original U- dimensional feature vector that has proven to be more useful in prediction accuracy.

DeepPSL [63] is a deep learning-based predictor that automatically learns abstract and high-level feature repre-sentations of human proteins through non-linear relations among broad subcellular locations. SubCons [64] uses a Random Forest Classifier (RFS) to combine four predic- tors collectively named MultiLoc2 [65], SherLoc2 [66] and CELLO2.5 [67] and LocTree2 [68]. SubCons integrates dif- ferent features from each predictor to design a multi-method prediction tool. CELLO2.5 [67] deals with determining phys- iochemical properties, i.e. compositions of di-peptide, amino acids, partitioned amino acids, and sequence compositions. LocTree2 [68] uses a cascading mechanism to determine cellular sorting. MultiLoc2 [65] participates in integrating the output of all these four classifiers and making SubCons fully functional. SherLoc2 [66] finally links UniProt IDs depending on PubMed. Combination-based predictors with mix-and-match approaches often result in better accuracy than individual predictors used to build SubCons.

LOCALIZER [69] predicts effector localization in plants while prioritizing effector targets for further evaluation. Gen- erally, plant-based predictors are functional on both host and pathogens. However, effectors exploit plants for the sake of entering organelles. In the majority of cases, effectors do not share sequence similarities with other existing pro- teins. Therefore, LOCALIZER plays a significant role in predicting whether a plant or effector protein can localize to multiple compartments. Plant-mSubP [70] is functional based on various hybrid features related to auto-correlation and quasi-sequence-order descriptors and dipeptide compo- sition (NCC-DIPEP) to attain better accuracy for multi-target localization. DeepLoc 2.0 [71] is multi-label subcellular lo- calization predictor that uses protein language models. It uses attention pooling of a protein sequence embedding, along with Multi-layer Perceptron (MLP) for predicting class probabilities and prediction accuracies.

CBOW [72] utilizes deep neural network based NLP method, and bi-LSTM, to accurately predict protein subcel- lular locations. It provides a pipeline that not only offers a high-throughput framework for linking biological entities from unstructured text but also facilitates the extraction of protein functional features. iLoc-Animal [73] is special- ized for animal proteins based on a multi-label K-nearest neighbour classifier while using sequence-based information. Multi-label predictors like these are capable of residing or transiting among two or more different subcellular locations simultaneously and are often known as multiplex proteins.

MFannot [74] is a tool capable of predicting protein lo- cations along with predicting DNA protein-coding genes. Its subcellular localization prediction for protein relies on pro- file Hidden Markov Models. This tool identifies incomplete protein models and then applies protein fusion methods to location information via HMMER. OrganelX [16] is a hy- brid predictor that uses two different approaches i.e. protein sequence embedding Unified Representation (UniRep) [75] and the Sequence-to-Vector (SeqVec) [76]. UniRep provides amino-acid embeddings that summarizes physicochemical properties. Whereas, SeqVec (Sequence-to-Vector) is based on context-dependent transfer-learning model ELMo which is a auto-regressive model. SeqVec uses ElMo as it allows processing of sequences of variable length. Also, it uses two layers of bidirectional LSTMs that introduce the context information. OrganelX also hosts two existing algorithms sub peroxisomal (In-Pero) [77] and sub-mitochondrial (In-Mito) [77]. These two predicting algorithms predict peroxisomal and mitochondrial proteins on OrganelX behalf. OrganelX along with In-Pero and In-Mito trains a model called Is-PTS that further uses logistic regression on the SVM scores.

### 2.2 Subcellular localization prediction tools for prokaryotes

Prokaryotes are organisms comprised of single prokaryotic cells without Endoplasmic reticulum, microtubules, perox- isomes, etc. Most prokaryotic predictors consider Gram- positive and Gram-negative bacteria for making subcellular predictions. PRED-LIPO [78] uses the Viterbi decoding algo- rithm and is based on the Hidden Markov Model method for the prediction of lipoprotein signal peptides of Gram-positive bacteria trained on experimentally verified proteins. LipoP [79] uses forward decoding, a method based on regular ex- pression patterns. MetaLocGramN [80] utilizes features from various other predictors namely PSORTb3 [81], PSLpred [82], CELLO [67], and SOSUI-GramN [83] to take maxi- mum advantage of all their combined strengths. It produces better prediction accuracy in comparison with predictors used individually.

There are few predictors that either target gram-negative bacteria or gram-positive bacteria. One such example is Gapped k-mer [84] which is a prediction tool for gram- negative bacteria. This tool uses the Peptide information ex- traction method with the amino acid composition of protein sequences and k-peptide information. However, SP Predic-tion [85] is for gram-positive bacteria only that achieve pre- diction by using signal peptides generated through attention- based neural networks (Transformer Model). It is a ma- chine translation model that generates SP (signal peptides) sequences, followed by identification and classification of the pathway used. This tool targets intracellular and extracellular locations both.

### 2.3 Subcellular localization prediction tools for viruses

As viruses are not actually cells. However, localization pre- diction usually refers to locations in their host cell, or in the virion. Subcellular localization of viral proteins is critical as they serve as biomarkers for viral infections. These kinds of predictions aid in the diagnosis of viral diseases and tracking treatment effectiveness. One such recent example is E-MuLA [20], which is an Ensemble-based multi-localized attention feature extraction network tool for Viral Protein Subcellular Localization. E-MuLA performance is checked against various state-of-the-art algorithms through rigorous comparisons with LSTM, CNN, AdaBoost, decision trees, KNN.

pLoc-mVirus is a deep learning-based subcellular localiza- tion predictor specifically for multi-location virus proteins. It incorporates curated GO information and has better results than iLoc-Virus [86]. iLoc-Virus uses sequential evolution information and is considered a powerful predictor for as- sessing the quality of multi-label predictors. Another recent tool is VIRify [87] uses virus-specific protein profile hidden Markov models. TIt is capable of identifying sequences from both prokaryotic and eukaryotic viruses along with detecting and classifying taxonomic ranges relevant to them.

### 2.4 Subcellular localization prediction tools for multi-categories

**Fig. 4:**
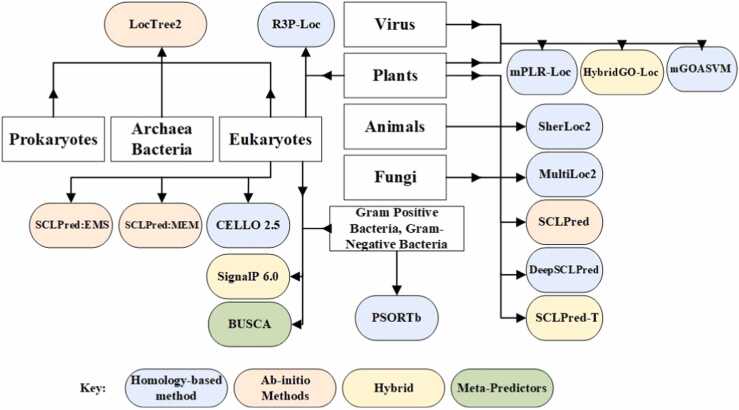
Tools for subcellular localization predictions supporting multi-categories.

Subcellular localization prediction tools for multiple cate- gories are tools that are capable of making predictions in different areas simultaneously. However, most of the predic- tors (as illustrated in Fig. 3) are designed to target one category at a time such as Human proteins, Animals, Algae or gram-positive, gram-negative bacteria. Whereas, multiple categories predictors (as illustrated in Fig. 4) are meant to target Eukaryotes, Gram-positive bacteria and Gram-negative bacteria together. Bologna Unified Subcellular Component Annotator (BUSCA) [88] integrates various computational tools to predict subcellular localization for globular and membrane proteins. Although BUSCA can produce predic- tions for Eukaryotes, gram-positive and negative bacteria, it is not yet designed for predicting proteins localized in lysosomes and peroxisomes.

R3p-Loc [58] extracts features from two ProSeq and ProSeq-GO databases to prove that these two databases func- tion similarly to using Swiss-Prot and GOA databases with less training time. mPLR-Loc [59] has an additional feature in prediction decisions to give probabilistic confidence scores for the prediction decisions (information about how the pre- diction decisions are made). mGOASVM [56] uses accession numbers, amino acid (AA) sequences, and a combination of both as input. Whereas, HybridGO-loc [57] adopts a hybrid approach to use GO term occurrences with the inter-term relationships by accessing GO frequencies in correspondence with the semantic similarity between them.

MultiLoc2 [65] detects the presence or absence of desired motifs via the MotifSearch Module integrated based on the amino acid composition. Sherloc2 [66] includes an additional text search module based on the PubMed abstract linked with the UniProt IDs. CELLO 2.5 [67] uses jury voting out of four votes given by amino acid composition, di-peptide com- position, partitioned amino acid composition, and sequence composition. Finally, SCLPred [89] is designed for mapping whole sequences (non-redundant sets of protein sequences) through a single functional class. SCLPred automatically compresses sequences into hidden feature vectors without considering resorting to predefined transformations.

Eukaryotic Subcellular Localization Prediction (SCL- Epred) [90] is based on an N-to-1 Neural Network Archi- tecture (N1-NN) similar to SCLpred. It is trained on tenfold cross-validation that mainly targets prediction of localization of proteins from least considered subgroups, i.e. Chroma- lveolates, Rhizaria, and Excavate supergroups (i.e. SAR- Excavates group) and SCL-Epred achieves better predictions within this area of choice. Another member of the SCLpred family of predictors is SCLpred-EMS [19], which specializes in eukaryotic protein prediction for the endomembrane sys- tem and secretory pathway versus all other amino acids se- quences, and is based on Deep N-to-1 Convolutional Neural Networks. A summary of recent protein subcellular localization prediction tools is given in Table 1.

**Table 1:** A summary of subcellular localization prediction tools.

Tools	Year	Categories	No. Locations/ Features Prediction (approx)	Methods/Models
E-MuLA [20]	2024	Virus	06	LSTM, CNN, KNN
ML-FGAT [91]	2024	Human Gram-positive bac- teria, Gram-negative bacte- ria, Virus, plant	19	Graph CNN
MFannot [74]	2023	Eukaryotes (any)	01	HMM, covariance
OrganelX [16]	2023	Eukaryotes (any)	02	SVM, Multi-class classifiers
Graph-BERT [92]	2023	Eukaryotes (any)	03	PPI Network, SeqVec
AdaPPI [93]	2023	Eukaryotes (any)	05	PPI Network
VIRify [87]	2023	Virus	00	protein profile HMM
SDN2GO [94]	2023	Human, Yeast	06	CNN, BiGRU
Mit Protein Trans- former [95]	2023	Eukaryotes (any)	04	Transformer Model, Deep CNN
DeepLoc 2.0 [71]	2022	Eukaryotes (any)	10	transformer language model
GraphLoc [21]	2022	Eukaryotes (any)	08	Graph CNN, BiLSTM
MSTLoc [96]	2022	Human	08	DNN, Deep Imaging-based Approach
PBLoc [97]	2022	Eukaryotes (any)	10	FFNN, BiGRU
SignalP 6.0 [98]	2022	Archaea, Eukaryotes, Gram- positive and Gram-negative bacteria	16	ProtTrans Models
MPSLP [99]	2022	Eukaryotes (any)	05	Self-attentionmechanism, DCNN, RAkEL
TooT-SC [100]	2022	Eukaryotes (any)	12	BERT
ProtPlat [101]	2022	Eukaryotes, Gram-negative, Gram-positive	10	BERT
SecProCT [102]	2021	Human	02	Transformer based models, CapsNet block
MULocDeep [103]	2021	Eukaryotes (any)	10	Self-attentionmechanism, LSTM
TripletProt [104]	2021	Human	14	FFNN
DeepPSP [104]	2021	Eukaryotes / Human	01	CapsNet block, Bi-LSTM block
AlexNet,VggNet, Xception, DenseNet [105]	2021	Human	07	DNN, Deep Imaging-based Approach
Transformer-XL, XLNet [106]	2021	archaea, bacteria, eukarya, viruses	12	ProtTrans Models
ProtTrans [107]	2021	archaea, bacteria, Eukary- otes, viruses	12	BERT
DeepPSL [63]	2021	Human	10	SAE networks
In-Pero [77]	2021	Eukaryotes (any)	02	PSSMs, SVM, Bi-LSTM
iDeepSubMito [18]	2021	Eukaryotes (any)	04	CNN, BiLSTM, ELMo
PSLOP [22]	2021	Eukaryotes (any)	33	BiLSTM, BiRNN, PSSM
SCLpred-MEM [19]	2021	Eukaryotes (any)	02	Deep N-to-1 CNN
PSORTdb 4.0 [108]	2021	Gram-positive,Gram- negative bacteria	07	Pattern matching
PSORTm [109]	2020	Prokaryotes and Archaea, Bacteria	07	HMM
SP Prediction [85]	2020	Gram-positive bacteria	10	ANN
ImPLoc [110]	2020	Human	06	FFN
CTM-AECA-PSSM- LDA [17]	2020	Eukaryotes (any)	01	Position-SpecificScoring Matrices (PSSMs), SVM
IM-Psepssm [111]	2020	Eukaryotes (any)	01	PSSMs, SVM
FSVM-KNR [112]	2020	Eukaryotes (any)	33	PSSMs
DCNN [113]	2020	Eukaryotes (any)	07	Ant-colonyoptimization, RAkEL
GasPhos [114]	2020	Human	06	Ant-colony optimization
DeepMito [115]	2020	Eukaryotes (any)	01	Deep CNN
ML-RBF [116]	2020	gram-positive bacteria and virus protein	10	Position-SpecificScoring Matrices (PSSMs)
HumLoc-LBCI [41]	2020	Human	16	GO
Plant-mSubP [70]	2020	Plants	14	K-mer Compositions
pLoc_Deep- mAnimal [117]	2020	Animals	20	BLSTM
SCLpred-EMS [19]	2020	Eukaryotes (any)	18	Deep N-to-1 CNN
BUSCA [88]	2019	Eukaryotes, Gram-positive bacteria,Gram-negative bacteria	21	Hybrid
SeqVec [76]	2019	Eukaryotes (any)	10	BERT, ELMo
TargetP 2.0 [118]	2019	Eukaryotic, Plants, Fungi	04	BiLSTM
GCGRandNSI [119]	2019	Eukaryotes (Human)	06	K-mer Composition, SVM
Gapped k-mer [84]	2019	Gram-negative	06	K-mer Composition
pLoc-mGneg	2018	Gram-Negative bacteria	08	MLT
SubCons [64]	2017	Human	11	RFC, Ensemble Method
DeepLoc [71]	2017	Eukaryotes (any)	10	FFN, BLSTM, A-BLSTM, Conv A-BLSTM
LOCALIZER [69]	2017	Plants	03	HMM
pLoc-mGpos [120]	2017	Gram-Positive bacteria	04	ML-GKR
iLoc-mGpos [120]	2017	Gram-Positive bacteria	04	KNN
pLoc-mVirus [121]	2017	Virus	06	ML-GKR
mGOF-loc [122]	2016	Human	37	PSSMs
mPLR-Loc [59]	2015	Virus and Plants	12	MLR
R3P-Loc [58]	2014	Eukaryotes and Plants	22	RP, ERR
HybridGO-Loc [57]	2012	Virus and Plants	12	GO, SVM

Key:

LSTM: Long Short-Term Memory Networks, CNN: Convolutional Neural Network, KNN: K-Nearest Neighbour, HMM: Hidden Markov Model, SVM: Support Vector Machine, PPI: Protein-Protein Interactions,

BiGRRU: Bidirectional Gated Recurrent Unit, BiLSTM: Bidirectional Long Short-Term Memory Networks, BiRNN: Bidirectional Recurrent Neural Network, DCNN: Deep Convolutional Neural Networks,

RAkEL: Random k-labelsets, FFNN: Feed-Forward Neural Network, BERT: Bidirectional Encoder Representations from Transformers, PSSMs: Position-Specific Scoring Matrices, ANN: Artificial Neural Network, ELMo: Embeddings from Language Models,

MLT: Multi-Label Theory, RFC: Random Forest classifier, MLR: Multi-Label Predictor,

MLGKR: Multi-Label Gaussian Kernel Regression, ERR: Ensemble Ridge Regression Classifier, CBOW: The Continuous Bag of Words.

Furthermore, SCLpred-MEM [123] is designed for pre- dicting membrane and non-membrane proteins. Among all other SCLpred family of predictors that are annotation-based, only SCLpredT [124] is template-based. In this case, the N-to-1 architecture is modified to accommodate template information, alongside its average quality/similarity to the query protein. DeepLoc [71] is based on convolutional motif detectors (a filter designed to position-specific scoring matri- ces for sequences) and selective attention to sequence regions (identifying protein regions) for making suitable predictions. SignalP 6.0 [98] is a tool that functions based on Signal peptides (SPs) that are short amino acid sequences. Sig- nal peptides are usually predicted through sequence data. SignalP 6.0 uses bidirectional encoder representations from transformers (BERT) protein language models (LM). BERT LM is available in ProtTrans (pretrained language models for proteins). BERT is based on transformers which is a deep learning model in which all output elements are connected with input elements and weightings between them are dy- namically calculated based upon their connections. Whereas, TargetP 2.0 [118], is a tool that predicts features embedded in the sequences and identifies N-terminal sorting signals. It detects the strongest signals and derives the classification based on that. It also uses BiLSTM to calculate the multi-attention matrix.

## 3 CHALLENGES AND RESEARCH GAPS

Based on the above-mentioned comprehensive study of sub- cellular localization prediction tools, various research chal- lenges and gaps are identified that require further attention and effort from researchers working within the field. As illustrated in Fig. 3, Fig. 4, homology-based methods (template-based) are generously present that predict various locations with better prediction accuracy. However, ab-initio- based predictors are quite less and the accuracy of their predictions is comparatively less than homology-based meth- ods, hybrid and meta-predictors. Ab-initio-based predictions (only amino acid sequence-based) require considerable atten- tion to cover the significant localization gap of unannotated proteins.

As illustrated in 2, the designing of prediction tools during the last 10 years has considerably grown. However, it can be witnessed that the homology modelling method is the most popular approach that provides advantages relevant to homology modelling with a simple algorithm, fast prediction speed, and high accuracy for proteins that have structure- known homologs [125]. However, the challenging part is that it strongly depends on the template structures, which means that it cannot predict structures of proteins whose homologs’ structures have not been determined [126]. Unlike homology modelling, trans modelling does not depend on the known protein structures but generates the 3D structure of a target protein only based on the established laws of physics (quantum mechanics) [127]. Available ab initio prediction methods tend to have low prediction accuracy in comparison with homology-based prediction methods [128]. Homology-based prediction methods achieve better percentages due to template-based identification of protein location. However, locating protein from sequences only is a challenging as well as tedious task to accomplish [129].

Even though trans modelling does not rely on the known protein structures, it has the possibility of finding new protein structural types [130]. Still, these methods are challenging in terms of free energy function i.e. accurate calculation of free energy would involve solving the Schrödinger’s equation, which requires a huge amount of calculation that is mostly not affordable. Secondly, the possible conformational num- ber of a protein with several hundred amino acids is estimated to be about 10^300^ [131]. However, great signs of progress have been made in conformational search algorithms, as well as computing power and storage space. Even though there are a lot of subcellular prediction tools and methods available, the Protein sorting process is still very complex and not yet completely understood [132]. Only a small portion of proteins have clearly identifiable sorting signals in their primary sequence [133].

**Fig. 5:**
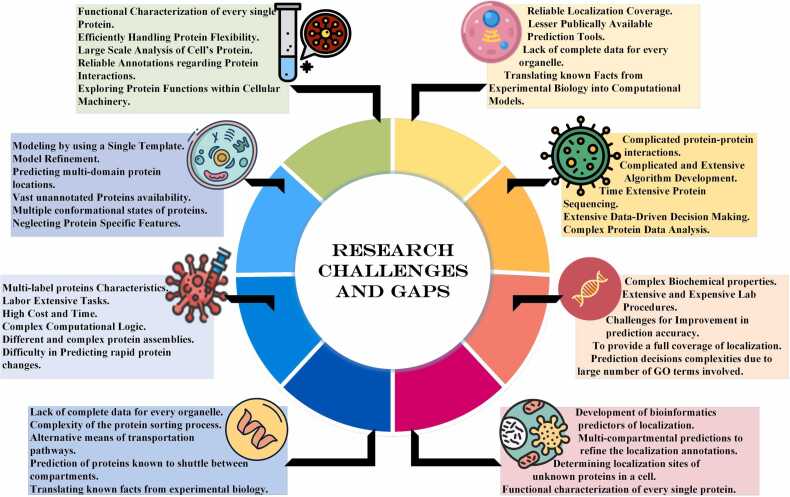
Dendrogram for subcellular localization predictions challenges and research gaps.

As illustrated in Fig. 5, there exists a high possi- bility that homologous sequences share the same struc- ture/function. However, they might not belong to the same subcellular localization. This factor results in wrong clas- sifier training while stopping it from correctly annotating the subcellular localization [134]. Also, composition-based approaches are mostly confined to amino acid composition- based features that are eventually not representative of other important aspects for the prediction [135]. Another critical challenge linked to integrated-based methods is their ability to incorporate various features but eventually suffer from over-fitting problems [136]. Apart from these challenges, using redundant training sets, overestimating the prediction performance, lower accuracy score, unconscious bias in fea- ture selection, mispredicting the query point and human errors still create troubles for bio-informatics users [137].

Automating prediction processes with higher accuracy is a challenging domain in computational biology as tradi- tional approaches are labour-intensive and extremely time- consuming [138]. Generating reliable and automated meth- ods capable of overcoming computational difficulty still needs various advancements for better prediction results. Fig. 5 also indicates that structural or sequence homology can be inaccurate since proteins with significant amino acid sequence identity possibly can have different functions [139]. Interestingly, some of the proteins also carry two biochemical or biophysical functions that add more complications to the prediction process. while few of the proteins can have no functions at all [140]. There might be a case that proteins claimed to have no functions are not fully characterized as others for now.

Challenges related to protein data pose serious concerns for subcellular localization predictions. Firstly, data quality is compromised due to the limited availability of high-quality labeled datasets for training predictive models, result in hin- dering prediction accuracy. Additionally, biased predictions are challenging due to imbalanced datasets, where certain subcellular locations have more examples than others, lead- ing to biased predictions. Secondly, predicting localization for novel proteins without experimental data is challenging and requires robust and complex computational methods. Extending prediction models to handle limited experimental data or different cellular architectures is also considered difficult in subcellular localization predictions.

## 4 Conclusion

We have concluded that despite numerous challenges, tools for protein structure prediction and design have advanced considerably in the recent decade. This article analyzed and explored various subcellular localization predictors based on their domain, methodologies adopted, accuracy achieved, and the number of locations successfully targeted by the predictors. A very well-directed and broad-spectrum tools- based diagrammatic illustrations are given in the article that can potentially help the readers to decide the tools of their choice with up-to-date review. A brief discussion is added that covers all major categories (Eukaryotes, Prokaryotes, and Viruses) of subcellular localization prediction to facil- itate researchers and bioinformaticians with better choices for accomplishing subcellular localization predictions. Soon, the rapidly increasing amount of diverse experimental protein data and advancements in computational methods and tools that make use of these data may result in improved accuracy and reliability of subcellular localization predictions.

## CRediT authorship contribution statement

**Maryam Gillani**: Writing – review & editing, Writing – original draft, Resources, Project administration, Methodology, Investigation, Funding acquisition, Formal analysis, Data curation, Conceptualization. **Gianluca Pollastri**: Writing – review & editing, Writing – original draft, Supervision, Resources, Project administration, Methodology, Investigation, Funding acquisition, Formal analysis, Data curation, Conceptualization.

## Declaration of Competing Interest

We here by declare that we do not have any conflict of interest.
